# Exploring gender biases in ML and AI academic research through systematic literature review

**DOI:** 10.3389/frai.2022.976838

**Published:** 2022-10-11

**Authors:** Sunny Shrestha, Sanchari Das

**Affiliations:** Inspirit Lab, University of Denver, Denver, CO, United States

**Keywords:** machine learning, gender bias, SOK, inclusivity and diversity, artificial intelligence, recommender systems

## Abstract

Automated systems that implement Machine learning (ML) and Artificial Intelligence (AI) algorithms present promising solutions to a variety of technological and non-technological issues. Although, industry leaders are rapidly adopting these systems for anything from marketing to national defense operations, these systems are not without flaws. Recently, many of these systems are found to inherit and propagate gender and racial biases that disadvantages the minority population. In this paper, we analyze academic publications in the area of gender biases in ML and AI algorithms thus outlining different themes, mitigation and detection methods explored through research in this topic. Through a detailed analysis of *N* = 120 papers, we map the current research landscape on gender specific biases present in ML and AI assisted automated systems. We further point out the aspects of ML/AI gender biases research that are less explored and require more attention. Mainly we focus on the lack of user studies and inclusivity in this field of study. We also shed some light into the gender bias issue as experienced by the algorithm designers. In conclusion, in this paper we provide a holistic view of the breadth of studies conducted in the field of exploring, detecting and mitigating gender biases in ML and AI systems and, a future direction for the studies to take in order to provide a fair and accessible ML and AI systems to all users.

## 1. Introduction

Algorithmic fairness has been a topic of interest in the academia for the past decade. Due to widespread application of Machine Learning (ML) and Artificial Intelligence (AI) assisted systems and complicated process of implementation, it is often difficult to flesh out the details of an ML system and demonstrate if the automated decisions made by these systems are fair or not. Although, the fairness discussion within the realm of ML and AI is a recent development, discrimination has roots within human society. The unfair treatment toward the minority is well-documented in the data we have created over time. These historical biases trickle into the automated systems through the data. ML/AI systems trained on these data are able to pick the implicit biases exercised over the years, which we might not see at first glance. For example, advertisement algorithm showing more high-paying technical jobs to men than women.

Although there are a variety of biases present in the automated decision-making systems based on attributes like gender, race, sex, age, and others, in this paper our focus in on gender biases. Broadly in the context of ML and AI implementation, a model is gender biased if the model's performance and/or output is biased against a faction of population based on their gender. For example, in the research conducted by Buolamwini and Gebru we see that the facial recognition systems were highly inaccurate (more than 30%) when it comes to classifying the faces of women of color. In this ground-breaking paper, the authors further demonstrated that the model was most accurate for people who identified as male and of white skin tone (Buolamwini and Gebru, 2018). Gender bias has been studied extensively in Natural Language Processing (NLP) systems because this is the most visible form of gender bias (Sun et al., 2019). Especially, in widely used language translation systems ML/AI models assign pronouns to profession confirming to the gender stereotypes, for example the models automatically assign he/him pronouns to professions like doctors and pilots whereas it assigns she/her pronouns to nurses and flight attendants (Cho et al., 2021).

However, gender bias is most harmful when the bias is not as readily noticeable. Especially, in the systems of online recommendations, social programs, national defense, justice systems and policing, which implements ML/AI algorithm, when the decision made by the automated systems might be gender biased but there is no definite way to confirm. Additionally, the ML/AI systems usually use the binary concept of gender which does not reflect the real world, and completely ignores population who might not identify with the either male or female identity.

These revelations has led to even more research into gender bias detection and mitigation methods that could help the ML and AI models to prevent gender bias in automated decision making. The fairness debate although nascent is a widely accepted concept and there is continuous effort to mitigate these biases from both academia and the industry. The industry leaders like IBM has a dedicated code repository, AIF360 that encourages ML and AI developers to learn, utilize, and normalize the use of bias detection and mitigation methods within their models.

Still more effort and work is needed to prevent gender biases and make these applications more inclusive and fairer across all users. Although every scientific publication and research lends immense insight into ML and AI systems and pervasiveness of gender biases in the implementations, there is a need for a holistic account of all the work done in this area. A comprehensive review of all the research will give us the benefit of looking into the areas that are well-researched and the areas that might need more research. It also helps us understand the trend of gender biases within AI/ML systems.

### 1.1. Key contributions

To this end, in this paper we provide a comprehensive view of all the research conducted in the field of gender biases propagated by ML and AI systems in the many implementations. The Systematization of Knowledge (SOK) is an effective format to provide a unified view of several aspects of gender biases in different forms and stages of ML and AI application. Furthermore, a detailed analysis of academic publications will help researchers and ML/AI engineers to understand the gaps in research that needs more attention. In short, this paper aims to aid the ongoing research into gender bias in ML/AI systems by providing following key contribution:

- **Provide an overview different themes and topics explored in the ML/AI gender bias research papers**: There are many interesting and innovative concepts presented by different authors published in the gender bias research field. These ideas bring novel solutions and perspective to the issue of gender fairness within ML and AI communities. Hence, it is valuable to create a holistic account of these ideas as they can inspire further conversation and actions to prevent gender bias.
- **Discuss different bias detection and mitigation methods proposed in these papers for different ML/AI algorithms**: There are many gender-bias detection and mitigation methods proposed in the literature but there is little wide-spread application of these methods. Since many of these methods are provided within the context of specific type of algorithm, these solutions could remain hidden from researchers working on a separate set of algorithms. So, it is important to gather these concepts and recount these ideas as they can result in creating more solutions to a similar problem in a different ML/AI application. Additionally, an overview of all these proposed methods gives us a clue into algorithms and solutions that are most studied and others that are not.
- **Shed light into the less studied aspects of ML/AI gender bias research and provide an argument for the need of more attention to these less explored topics**: The ethical and legal aspect of these gender bias issues are seldom discussed. Similarly, we need more studies that collaborate with user community and field experts to get a good grasp on their perspective of gender bias in ML/AI systems.

Here, we would like to mention that there are prior literature reviews which conduct in-depth analysis of fairness in automated systems and provided detailed overview of bias mitigation and detection methods. For example, Caton et al. review different biases detected in automated systems based on sensitive attributes like gender, marital status, race, and disabilities (Caton and Haas, 2020). Whereas, Mehrabi et al. go even further and evaluate biases based on attributes like race, color, gender, national origin, religion, and so on (Mehrabi et al., 2021). Both of these papers focus more on the technical details regarding biases and methods proposed in the literature. Our study focus on gender biases while emphasizing on the societal impact such biases have. Additionally, we provide details from prior research related to this specific topic while detailing the solutions proposed and applied while emphasizing on the feasibility of these solutions. Thereafter, we conclude by providing analysis of the nature of gender bias and the potential harm of such biases in different automate system implementations.

In the following sections, we will describe our methodology for data collection and SOK in Section 2. We will discuss the major themes discovered in the publications in Section 3, we will detail the implications of these themes in the Section 4. Thereafter, we will outline the limitation of this work and the future extension of our work in Section 5. Finally, we will conclude the paper by providing the summary of the SOK in Section 6.

## 2. Methodology

We began our study by first looking at similar prior SOKs published in the field to better understand the methodologies of conducting a thorough literature review. We reviewed papers by Stowell et al. and Das et al. to understand the methodology of conducting a systematic literature review. Their research focused on mHealth intervention for vulnerable population, and phishing and authentication respectively (Stowell et al., 2018; Das et al., 2019). Drawing inspiration from these papers we have implemented following methods in our study: (1) keyword-based database search, (2) data screening and quality control: content screening based on paper's title, abstract and full text, and (3) data analysis.

This literature review is guided primarily by the following research questions:

- What is the current research landscape on gender specific biases present in ML systems and models?
- What technical solutions are proposed to detect, mitigate, or eliminate gender specific biases in prior research?

### 2.1. Database search

From our study into literature reviews, we found keyword-based search to be more effective way to gather relevant papers in a short amount of time. Our research goals motivated the keywords we have used in the database search. We identified that we wanted to limit our study to papers discussing “automated-decision making systems”, however because the term is verbose and ambiguous, we settled on keywords such as “*machine learning or ML*”, “*artificial intelligence or AI*”, and “*automated system*”. We also wanted to narrow our research to gender related biases discovered in these systems, so we added following keywords in the search: “*gender*”, “*algorithmic bias*”, “*gender bias*”, “*gender bias in automated system*”, and “*gender bias in machine learning*”. We used various combinations of these words using “+ ”or logical connectors “AND”and “OR”.

In this study, we have used the Publish or Perish software to collect the papers. This is because this software allows us to conduct search into multiple digital libraries at once and also allows us to filter results based on publication year, titles, and other criteria. With the help of this software we were able to conduct keyword-based search across digital libraries including, Google Scholar, ACM Digital Library and IEEEXplore. At the time of our initial search (December 2021), searching in Google Scholar required no prior registration. We limited our search to the publications from year 2010 up to 2021. We added this year restrictions because we believed any research prior to 2010, will not reflect the current developments in the field. From the search we gathered 192 papers for the review.

### 2.2. Data screening and quality control

Once we had all the papers, we manually went through the papers to further refine the corpus. We created following exclusion criteria to refine the papers we had collected:

- We excluded the paper if the paper was not written in English given the primary evaluation done in the same language.
- We removed the paper if the full text of the paper was inaccessible, behind a paywall or had loading errors. We contacted the authors in that case, and we kept the paper in our list if we obtained those papers.
- We excluded the paper if the paper was incomplete or retracted, or not published on peer-reviewed journals and/or conferences.
- We put a time constraint in the inclusion of the papers and analyzed those papers published on or after 2017. We intentionally put this criterion to evaluate the recently published work in the last 6 years.

When we looked into the papers during this excluding exercise, we found that most of the papers published prior to 2017 were not relevant to the topic at hand: gender bias in automated decision-making systems. Also, few papers that were published prior to 2017 and had relevant information had additional or updated work published after 2017, hence we arrived at the final exclusion criteria listed above.

We implemented the exclusion criteria in three phases of screening. First, we reviewed just the title, keywords, and abstracts of the paper. Then, we reviewed the full text of the papers and created a codebook based on the paper's focus. Finally, we analyzed the methods and implications of the papers in a detailed manner to arrive at the final review corpus. We removed a total of 19 papers from our results based on the criteria mentioned, thus resulting in 173 papers. Next, we excluded papers that showed up twice which further reduced the number of papers in the corpus to 154.

#### 2.2.1. Title and abstract screening

We further screened the remainder of 154 papers based on their titles and abstracts. In this step, we carefully reviewed the collection of papers to makes sure they had relevant keywords which includes words like “gender”, “bias/biases”, “machine learning”, and/or “artificial intelligence”, within the titles and abstracts. Based on the presence of these keywords, we classified papers into two categories: “relevant”, “some relevancy”, and “irrelevant”. During this processing we excluded more papers that fell into irrelevant category, resulting in total corpus count of 149.

#### 2.2.2. Full paper screening

**Figure 1:**
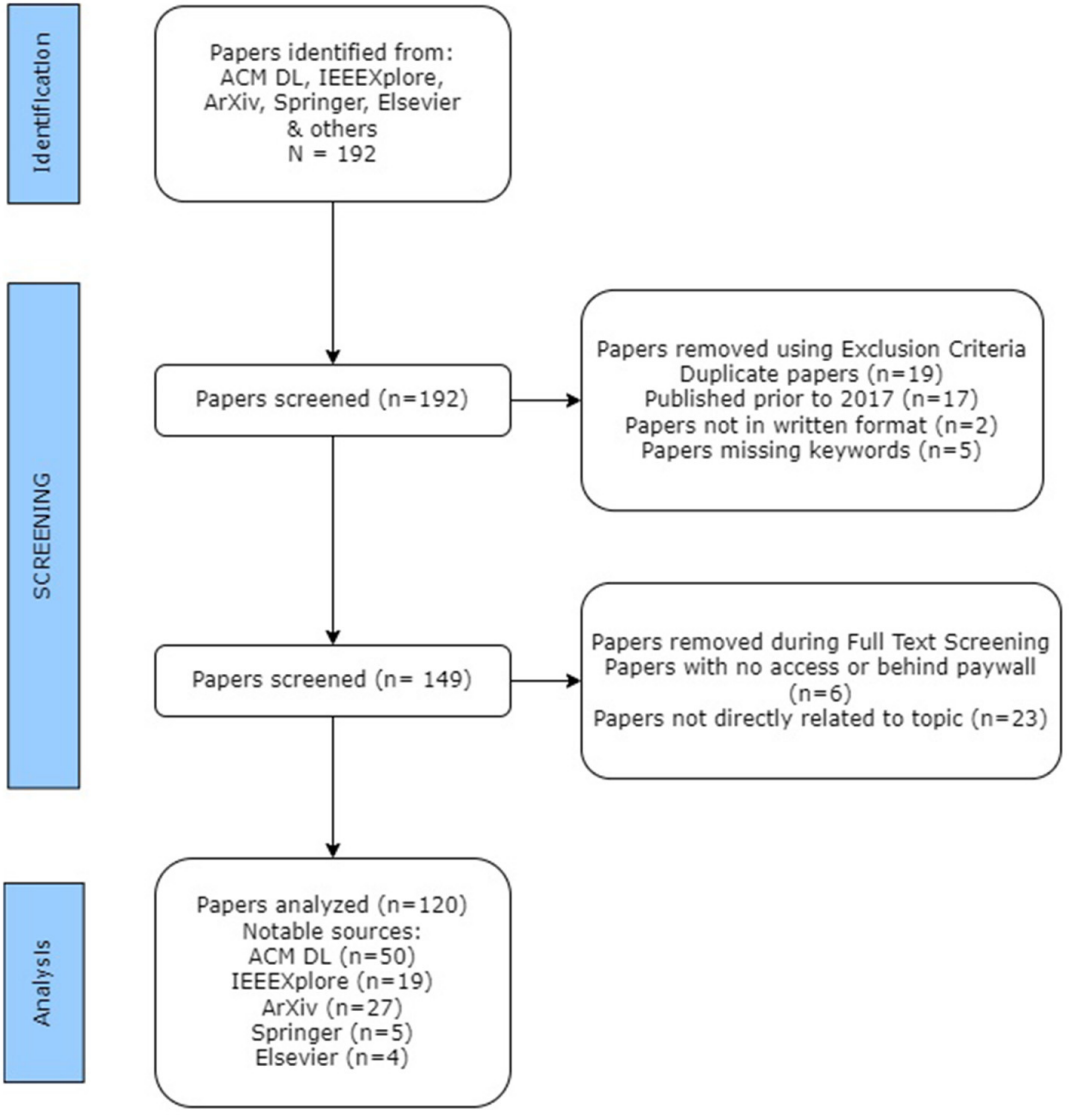
A visual illustration of paper screening methodology used.

In this phase, we conducted a quick review of the full text of the 149 papers mainly focusing on the study methodology, implications, algorithms explored, and solutions proposed. In this step we removed 6 papers from the corpus because the full-text version of these papers were inaccessible or behind a paywall. We also further excluded 23 papers because they did not directly discuss about gender biases in the automated decision-making systems. Thus, we ended up with a total of 120 papers as summarized by the diagram (Figure 1).

### 2.3. Analysis

**Figure 2:**
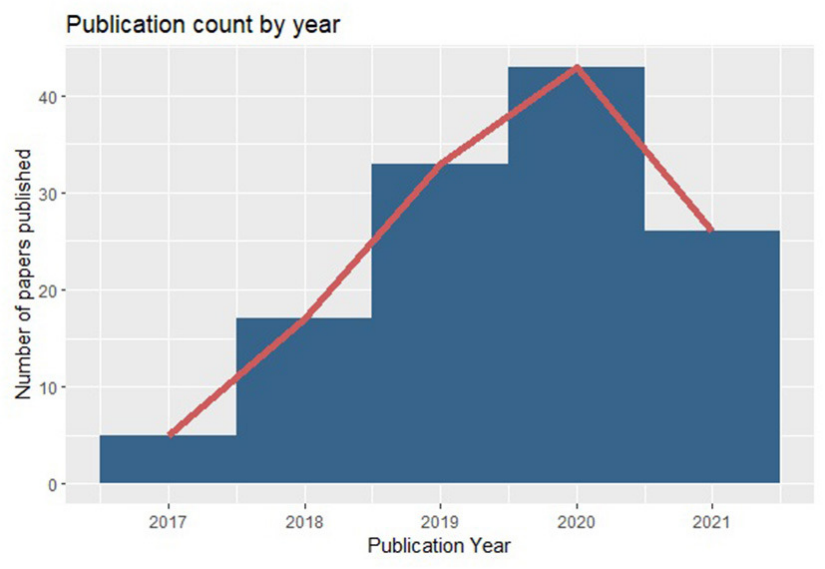
The number of papers of published for gender bias in automated systems based on publication year.

After the screening, we conducted a detailed review of 120 publications focusing on the algorithms explored in the paper, methodology followed, and solutions proposed to detect and/or mitigate gender biases in the automated decision-making systems. We also further analyzed the papers based on the publication year to trace the research trend on this topic over the years. As demonstrated in the Figure 2, there has been drastic increase in research into gender bias in automated decision-making systems over the years. We also created a codebook to categorize papers into different groups based on the focus of the paper. Details of this codebook can be viewed on this Table 1.

**Table 1:** Distribution of papers collected for this review based on the focus of paper.

**Focus of papers**	**Paper count (percent)**	**Sub-themes**
Gender bias analysis	48 (39.7%)	AI [4], NLP [14], Facial Data Analysis [8], Legal & Ethical Implication [9], Recommender Systems [3], Healthcare & Medicine [2], Policy & Government [2], Search & Ranking [2], Marketing [1], Automated Systems [1], Automated Recruitment [1]
Mitigation methods	34 (28.1%)	NLP [14], Facial Data Analysis [8], Recommender Systems [3], Classification [2], Legal & Ethical Implication [1], Marketing [1], NA [1]
Detection methods	19 (15.7%)	NLP [11], Facial Data Analysis [3], Automated Recruitment [2], Individual Fairness [1], Unwanted Associations [1]
User studies	8 (6.6%)	NLP [2], Facial Data Analysis [1], Legal & Ethical Implication [1], Search & Ranking [1], Recommender Systems [1], Others [2]
Case studies	5 (4.1%)	NLP [2], Legal & Ethical Implication [1], Classification [1], Recommender Systems [1]
Literature reviews	7 (5.8%)	NLP [3], Facial Data Analysis [1], Healthcare & Medicine [1], Search & Ranking [1], Bias Mitigation Frameworks [1]

## 3. Results

### 3.1. Gender bias in literature

Forty-eight out of 120 papers collected for this study discussed about the presence of gender bias in a variety of ML and AI applications. The diverse fields studied in these papers show the varied applications automated decision-making systems have and, it helps us realize the severity of the gender fairness issue. It also demonstrates that unintentional bias can have drastic effects on minority populations.

Fourteen papers discussed **Natural Language Processing (NLP)** systems and the presence of gender biases in these systems. Researchers studied NLP algorithms like Word Embeddings, Coreference Resolution and Global Vector of Word Representation (GloVe). In these papers authors discuss the presence of inherent bias in the human languages which is then codified into the ML and AI NLP operations through the data. Here, NLP operations refers to functions like embeddings, coreference resolution, dialogue generation, machine translation, text parsing, sentiment analysis, hate speech detection, and so on (Sun et al., 2019; Blodgett et al., 2020). Chen et al., look across nine human languages, including English, Spanish, Arabic, German, French, and Urdu, and find gender bias in gendered nouns for profession words (Chen et al., 2021) in Word Embeddings. In another paper authors Guo and Caliskan, explore the intersectional bias present in English static Word Embeddings (Guo and Caliskan, 2021). They find that women of African American and Mexican descent were most biased against because of their racial and gender identity. Similarly, authors also look into machine learning models and study the effects of gender biases in these applications (Stanovsky et al., 2019; Prates et al., 2020). Gender bias has also been detected and studied in application that predict a person's profession (Santana et al., 2018; De-Arteaga et al., 2019) and gender (Krüger and Hermann, 2019). Other NLP applications discussed in these papers that are affected by the gender bias are sentiment analysis (Folkerts et al., 2019), emotion identification (Manresa-Yee and Ramis, 2021), and customer review analysis (Mishra et al., 2019). Two papers discuss the representation of women in audio-visual medium, these papers discuss how a biased system can affect the gender-equality movement by presenting women in gender normative fashion. Gutierrez points out that the Google image search results for powerful profession like CEO, news reporters or movie directors lack women representation (Gutierrez, 2021). Similar paper by Singh et al. also highlights the issue of lack of women representation in occupations images in various digital platforms (Singh et al., 2020).

The **Automated Facial Analysis, image classification, and recognition algorithms** is the second most studied ML application in these papers. The research by Buolamwini et al. is a pioneering paper that shed light into algorithm fairness in automated systems was also conducted on Facial Recognition Technologies(FRT) Buolamwini and Gebru (2018). Srinivas et al. also conduct gender bias analysis on off-the shelf and government prescribed FRTs and found these systems to have biases even though the creators claim otherwise (Srinivas et al., 2019). Biases affects FRTs by affecting its performance accuracy creating mis-labeled or mis-classified faces for minority population (Krishnan et al., 2020; Balakrishnan et al., 2021; Manresa-Yee and Ramis, 2021; Raz et al., 2021).

Automated decision-support systems are prolific in the field of **advertisement, marketing, and recruitment** systems. Howcroft and Rubery discuss the effects of gender bias in the labor markets in disrupting social order and point out the need to tackle these biases from outside-in (*fixing the issue in the society before fixing the algorithm*. They discuss how implicit biases of the users, rooted in our social norms and habits, feed into these biased systems to create a regressive loop (Howcroft and Rubery, 2019). Another paper by Shekawat et al. discuss the presence of gender bias in ad-personalization applications that expose users to biased advertisements continuously through their devices (Shekhawat et al., 2019). In similar vein, Raghavan et al. present the legal implication of recruitment systems that are gender biased (Raghavan et al., 2020).

Five papers talk about the presence of gender bias in **recommender systems** and, **search and ranking** algorithms. In these papers too, we see the authors point out biases in the systems and how it affects our lives on the ground-level. Lambrecht and Tucker study the tailored job listings, which based on applicants' gender, present different job opportunities to different applicants. The jobs shown to women were discriminatory in nature as they were shown far fewer STEM ads than men (Lambrecht and Tucker, 2019). Another paper by Tang et al. replicate this study but provide an interesting insight into the implicit biases held by the applicants. In this paper, they demonstrate that job applicants are also affected by and affirm to the gender stereotypes, i.e., men tend to apply for more technical and ambitious jobs as compared to women (Tang et al., 2017). This paper further confirms theory presented by Howcraft et al. that the issue of gender bias is rooted in society and requires outside-in approach. Furthermore Wang et al. goes deeper and demonstrates how the implicit bias in the users interact with the biased systems creating a regressive loop, for example a biased system shows a gender-stereotypical job listing to applicants and applicants perpetuates this by selecting from these jobs instead of searching for non-stereotypic listing (Wang and Chen, 2021). Finally, Shakespeare et al.'s paper study the presence of gender bias in music recommender systems and shows the effect of these biases (Shakespeare et al., 2020).

Some papers also delve into the presence of gender biases in **AI & robotics** technologies. For example, some papers look into different specific incidences of gender bias in justice systems, medical robots, and self-driving cars (Howard and Borenstein, 2018; Amershi et al., 2019; Brandao, 2019). Lopez et al. present the existence of implicit gender bias in virtual reality where the users' bias affect the virtual avatars, they tend to choose (Lopez et al., 2019). Righetti et al. analyze the significant consequences of a biased model and argue for the importance of proper legislation and multidisciplinary mitigation approach to prevent such biases in AI and robotics (Righetti et al., 2019).

An interesting paper by Crockett et al. explored the gender bias effect on deception detection systems that uses Non-Verbal Behavioral cues exhibited by people and predicts if the subject is deceptive. Although they didn't find any significant effect of subject's gender on the prediction accuracy, they argued that the classifiers used to detect deception should be trained separately for different genders because that tends to work better for either gender than a one-size fits all approach (Crockett et al., 2020).

There were only two papers that studied the gender bias in automated decision-making systems used specifically for **governing and policymaking** purposes. The paper by Ester Shein with the help of poverty attorney highlights the ground reality of AI decision-makings in human social programs. The paper points out that although AI automates the systems faster and efficient, it might not necessarily be accurate. Due to the nature of human-services programs the fairness of these systems is crucial in these systems and the cases that comes across these systems require a nuanced solution which AI systems are not capable of (Shein, 2018). Likewise, Hicks demonstrate the effect of gender bias in government identification card issuing algorithms. It highlights the lack of representation of the non-binary and queer community in automated decision-making systems (Hicks, 2019). This is one of the few papers that advocates the need of representation of LGBTQ+ population in automated decision-making models.

Some papers study the existence and effects of gender bias in automated decision-making systems used in **medicine and healthcare** operations. Narla et al. touch upon the need to prevent gender biases in skin cancer detection algorithm (Narla et al., 2018). In a similar vein, Paviglianiti et al. focus on medicinal devices specializing in Vital-ECG for predicting cardiac diseases (Paviglianiti and Pasero, 2020).

Surprisingly, we found nine papers that expanded on the **legal and ethical implication** of gender biases in automated systems (Katell et al., 2020; Fleisher, 2021). This includes a paper by Koene et al., which is a work-in-progress paper regarding an IEEE industry standard to prevent algorithmic biases (Koene et al., 2018). Similarly, a paper by Karimi et al. discusses the presence of gender bias in criminal recidivism and highlights how a biased system affect female prisoners (Karimi-Haghighi and Castillo, 2021). Raghavan et al. looks into the legal implication of recruitment using a biased automated decision-making system (Raghavan et al., 2020).

Some papers describe the implication of gender biases from an ethical perspective. In their papers, Bird et al. and Glymour and Herington provide a comprehensive view of different types of biases based on the scope of the errors (Bird et al., 2019). Glymour and Herington also measure the severity of these biases and lay out the implication of these biases (Glymour and Herington, 2019). Gilbert and Mintz demonstrate the relationship between machines, humans, and data and show the impact of human cognitive bias in machine learning pipeline (Gilbert and Mintz, 2019). Moreover, the paper by Donnelly and Stapleton shows how a gender-biased system reinforces gender bias and can harm the marginalized population. This is an interesting paper as it focuses on the importance of creating a fair system that does not inflict discrimination against minorities (Donnelly and Stapleton, 2021). Finally, Fleisher et al. and D'Ignazio present the concept of individual fairness (Fleisher, 2021) and participatory design (D'Ignazio et al., 2020) to remediate gender bias from algorithmic systems.

A total of nine papers reviewed the gender bias issue in **Recommender and Search Engine Optimization(SEO)** algorithms. Authors studied bias in music recommender systems (Shakespeare et al., 2020; Melchiorre et al., 2021), career recommendation systems (Wang et al., 2021) and both SEO and Recommender systems in general (Novin and Meyers, 2017; Otterbacher et al., 2018; Geyik et al., 2019; Baeza-Yates, 2020; Boratto et al., 2021; Wang and Chen, 2021). One interesting paper by Howard et al. looked into specific incidences of gender biases in AI and robotics. For example, incidents of gender biases in justice systems, medical robot, and self-driving car (Howard and Borenstein, 2018).

### 3.2. Bias mitigation methods and frameworks

Thirty-four out of 120 papers propose different gender bias mitigation methods and frameworks. Algorithmic bias are usually prevented or mitigated by manipulating the source of the bias. In most cases the source is either the training corpus or the algorithm itself. Based on the phase of the training when the model designers introduce the intervention there are three different types of algorithmic bias mitigation (Feldman and Peake, 2021):

- Pre-Processing: In these methods, the intervention is introduced before the training starts. For example, data manipulation/augmentation, creating a checklist to vet the algorithm/data, targeted data collection are some of the tasks that can be done to prevent any unintentional bias ahead of time.
- In-Processing: In these methods, the intervention occurs during the model training phase. Adversarial learning is popular debiasing method that falls under the in-processing methods as the correction or debiasing occurs while the model is training. Applying corpus level constraints, relabeling the data are also some examples of this type of debiasing.
- Post-Processing: These mitigation methods are applied post training and are the most easily applicable methods among all three types of mitigation methods. Posterior regularization and Calibrated Equalized Odds fall under this category. This technique attempts to rectify the outcomes while minimizing errors.

Additionally, researchers Bender and Friedman present three broad categories of algorithmic biases based on their origin (Bender and Friedman, 2018),

- Pre-existing biases: these stem from biased social norms and practices. These biases get introduced into the ML systems through data.
- Technical biases: these are of technical nature and thus are introduced into ML and AI systems when the creators implement certain technical constraints and decision.
- Emergent biases: these are biases caused when ML system trained for a specific purpose is implemented for a different goal. For example, a FRT trained for Caucasian population when implemented on Asian population will tend to perform poorly thus creating bias.

In the following paragraphs, we discuss the mitigation methods that address specific methods that target pre-existing biases or data bias and technical biases or algorithmic bias.

#### 3.2.1. Data bias mitigation

Bender and Friedman suggest creating and maintaining data statements as a professional practice can reduce unwanted bias in the ML modeling. Here, by data statements they mean providing pertinent information on the dataset that is going to be used to train the ML models. By understanding the type and characteristics of the dataset, ML model creators and users will be able to gauge the prediction quality of the ML model and its appropriate application (Bender and Friedman, 2018). For example, if the data collected is not representative of the general population (minorities missing) or unbalanced (over or under representation of certain population), data statements provide such information on the data so that algorithm designers could use this information to proactively implement bias mitigation methods. Cramer et al. recognize these biases and thus propose a quite simple, yet effective method to tackle gender biases. They introduce the idea of using a checklists and ML/AI engineers getting acquainted with the world in which the model is going to be implemented. This helps engineers pause and think of the outcomes they would want to see instead of getting down to coding with little thought (Cramer et al., 2018) of the eventual impact of the system on the users. Baeza-Yates and Courtland also emphasize the idea of understanding the context of model implementation and the data used for model training to prevent pre-existing/data biases (Courtland, 2018; Baeza-Yates, 2020).

Authors also propose a balanced dataset for ML model training as a solution to dealing with data bias Wang T. et al. (2019). Here, a balanced dataset means a dataset that is representative of all demographics and comprised of both minority and majority population in equal proportion. There are different ways to achieve a balanced dataset like collecting more data from minority population, creating augmented data for the minority population (Smith and Ricanek, 2020), or removing the majority population data for the model training. The Facial Recognition Technology (FRT) suffers from unbalanced dataset because the publicly available face datasets have comparatively less faces of minority population than majority population. To resolve this author Karkkienen and Joo propose a dataset comprised of 7 race groups: White, Black, Indian, East Asian, Southeast Asian, Middle East, and Latino (Kärkkäinen and Joo, 2019). Dass et al. apply a similar approach to create a comprehensive and representative dataset by using mugshots data for mixed race groups: Black Hispanic, White Hispanic, Black non-Hispanic and White non-Hispanic (Dass et al., 2020). Similarly, authors Wu et al. bring the issue of lack of representation of non-binary population in the ML facial classification models. To rectify this, they propose two new databases; a racially balanced dataset with a subset of LGBTQ+ population and a dataset that consists of a gender-inclusive faces for binary and non-binary population (Wu et al., 2020).

Another interesting method proposed in these papers is Counterfactual Data Augmentation (CDA) to mitigate gender biases in Natural Language Processing (NLP) models (Maudslay et al., 2019; Lu et al., 2020). CDA is a method of data manipulation in which alternative version of the present data is added into the corpus to overall balance the gender representation in the corpus. For example; if the corpus has overwhelmingly high proportion of statements associating male gender with the profession *doctor*, like: “***He*** *is a doctor*”or “*The doctor provided* ***his*** *expert advice*”, then the counterfactual data will be created and added to the corpus like:“***She*** *is a doctor*”or “*The doctor provided* ***her*** *expert advice*”. This way in the overall corpus the gender representation is balanced hence, making it less likely that the resulting model will have gender bias stemming from the training data. In their paper authors Maudslay et al. present the concept of direct and indirect bias present in NLP and argue that CDA or a version of CDA can tackle both of such biases. In their solution they propose substitution of augmented data instead of blind addition, to maintain the grammar and discourse coherence, and bipartite graph matching of names as a better CDA approach (Maudslay et al., 2019).

#### 3.2.2. Algorithmic bias mitigation

Algorithmic bias mitigation measures involve updating the algorithm and the conditions on the algorithm in order to arrive at an optimal prediction. Unlike the data bias mitigation methods, these methods are very varied. However, there seems to be many post-processing debiasing methods.

Many of the authors demonstrate the use of adversarial debiasing techniques in various forms to get rid of gender bias from the ML/AI models. Adversarial learning is recognized as an effective measure to remove biases from ML models. Adversarial debiasing is an in-process debiasing technique in which the goal is to increase prediction accuracy while simultaneously reducing adversary's ability to predict protected values from the output prediction^1^. For example, in a credit-worthiness algorithm with gender as a protected attribute, the prediction of a person's worthiness should be highly accurate while also being ambiguous on the person's gender. In this type of learning the goal is to …“minimize the information extracted by the *encoder* that can be maximally recovered by a parameterized model, *discriminator*” (Hong et al., 2021). Case in point, in their paper Morales et al. utilize adversarial technique to remove sensitive information from the learning process which results into a fair and privacy-preserving facial analysis model. This learning strategy named as SensitiveNets removes sensitive information such as gender and ethnicity from the images while still being able to recognize and classify facial gestures or multi-modal learning (Morales et al., 2020). In similar vein, there are other paper that have used similar adversarial learning approach to debias visual recognition algorithm (Wang T. et al., 2019), dialogue systems (Liu et al., 2020) and facial recognition system (Dhar et al., 2020).

Some authors look into greedy algorithm to train their models to get the desired outcome. Here, the greedy algorithm delivers a fair model because the algorithm tries to maximize the fairness metric, as designed by the model creators, as it trains. In their paper, Barnabo et al. look into using three different greedy set cover methods and a linear programming method to get a representative professional team for labor match (Barnabò et al., 2019). Here, the greedy algorithm is trying to maximize the diversity of the professional team the algorithm picks while still meeting the labor match. For example, the workers picked by the algorithm to form a team should be able to complete the task at hand, the total labor cost of the team should be as minimum as possible, and the team should represent workers from all classes (like men, women and non-binary or workers from different races, ages as so on). Essentially, the algorithm will keep trying to put together a team of works for a work requirement that meets all the criteria mentioned above. Geyik et al. also use a post-processing greedy algorithm approach to mitigate gender biases in LinkedIn talent search. In their paper, they find that the debiased greedy algorithm yielded a representative sample 95% of the time in comparison to a non-debiased algorithm (Geyik et al., 2019). Farnand et al. also utilize greedy algorithm to mitigate gender bias in Influence Maximization problem, i.e., maximizing profits of an advertiser in a social network. The authors identify the statistical metric that denote fairness for example fair allocation of resource across groups. With the help of their greedy algorithm, they try to maximize this property of fair allocation thus delivering a fair system (Farnad et al., 2020).

Other methods proposed include using Equalized Odds processing technique, which is a popular method which is also included in the IBM AIF360 library (Singh and Hofenbitzer, 2019), using post-process regularization technique (Jia et al., 2020; Morales et al., 2020), Multi-task Convolution Neural Network Approach (MTCNN) (Das et al., 2018) and Langragian relaxation for collective inference (Zhao et al., 2017). One interesting method proposed by Feldman and Peake comprised of using a mix of different debiasing techniques like disparate impact remove (pre-processing method), adversarial debiasing (in-processing method) and calibrated equalized odds(post-processing method). We have summarized all of these different methods in the Table 2.

**Table 2:** Different bias mitigation methodologies proposed by the papers reviewed in this study.

**Algorithm family**	**Sub-category**	**References**	**Mitigation method**
NLP	Image Processing	Smith and Ricanek, 2020	Corpus Level Constraints
	Dialogue Systems	Liu et al., 2020	Adversarial Learning
	Voice Processing	Cramer et al., 2018	Checklists + Representative Data
	NA	Courtland, 2018	Algorithm Auditing
	Language Processing	Jia et al., 2020	Corpus Level Constraints + Posterior Regularization
	Language Processing	Bender and Friedman, 2018	Data Statements
	Word Embeddings	Prost et al., 2019	Scrubbing, Debaising and Strong Debiasing
	Word Embeddings	Wang et al., 2020	Double-Hard Debiasing
	Word Embeddings	Lu et al., 2020	Counterfactual Data Augmentation
	Word Embeddings	Maudslay et al., 2019	Counterfactual Data Augmentation
	Word Embeddings	Zhao et al., 2018	Gender Neutral Word Embedding
	Word Embeddings	Wang Z. et al., 2019	Representative Data
	Abusive Language Detection	Singh and Hofenbitzer, 2019	Equalized Odds processing
	NA	Courtland, 2018	Auditing Algorithms
	NA	Hitti et al., 2019	Gender Bias Taxonomy
	Advertising	Farnad et al., 2020	Greedy Algorithm
Automated facial analysis	Facial Recognition Task	Wang T. et al., 2019	Adversarial Debiasing
	Face Attribute Recognition	Kärkkäinen and Joo, 2019	Representative Data
	Gender Classification	Wu et al., 2020	Representative Data (Racial + LGBTQIA+)
	Facial Processing Technology	Dass et al., 2020	Representative Data + Human Annotated Data
	Automated Face Analysis	Das et al., 2018	Multi-task Convolution Neural Network
	Facial Classification Task	Molina et al., 2020	Data Augmentation
	Facial Recognition Task	Morales et al., 2020	Adversarial Regularizer
	Facial Recognition Task	Dhar et al., 2020	Adversarial Debiasing
	Automated Face Analysis	Katell et al., 2020	Algorithmic Equity Toolkit
Recommender system	NA	Baeza-Yates, 2020	Explore and Exploit Paradigm
	Music Recommender	Melchiorre et al., 2021	Resampling and Rebalancing Data
	Job Recommender	Geyik et al., 2019	Greedy Algorithm
	Job Recommender	Barnabò et al., 2019	Greedy Set Cover and Linear Programming
	Job Recommender	Vasudevan and Kenthapadi, 2020	Annotated Data + LiFT Framework
Object classification	NA	Chakraborty et al., 2020	Removing and Relabeling Data + FAIR_FLASH
	Collected Inference	Zhao et al., 2017	Langarian Relaxation
	Classification	Feldman and Peake, 2021	Disparate Impact Remover + Adversarial Debiasing + Calibrated Equalized Odds

### 3.3. Bias detection methods and frameworks

Detection of unwanted gender biases in a ML/AI model is as important as the mitigation of the biases. The detection frameworks allows to create benchmarks that model designers can use to vet these ML/AI models that will be implemented in far-reaching systems. In the paper collected for this review, nineteen out of 120 papers presented a detection mechanism or framework to assess the presence of gender bias in an algorithm.

A comprehensive detection framework proposed by Schwemmer et al. shows FRT systems like Google Cloud Vision, Amazon Rekognition, Microsoft Azure Computer Vision contain gender bias when compared against human coded dataset. All of the systems were able to accurately identify a person as women when the picture confirmed with feminine stereotype like hair length, makeup and so on. Some of the systems even labeled images with stereotypical feminine words like “kitchen” or “cake” when in fact nothing of that sort was present in the pictures. Furthermore, the authors point out that the identification of images is binary and there is no room for LGBTQ+ population in the prediction results (Schwemmer et al., 2020). Serna et al. present an InsideBias detection model that detects bias in deep neural network systems that classify and analyze facial data (Serna et al., 2021). Booth et al. also review gender bias in recruitment using video interview analysis. Their paper analyzes the bias present in image processing by utilizing psychometrics and affective computing (Booth et al., 2021). Pena et al. also look into bias in automated recruitment systems using FairCVtest, a gender bias detection framework that detects bias in training data (Pena et al., 2020).

The Winograd schema proposed by Levesque et al. has inspired some of the detection methods suggested in these papers. The Winograd schema operates on commonsense reasoning questions that is asked to the machine to test if the machine can distinguish the nuances of human languages as competently as most humans are able to. A Winograd schema usually involves twin ambiguous sentences that differ in one or two words, it requires a sense of the situation, reasoning, and intention of the sentence to identify the correct form of the sentence. It is used to test the commonsense reasoning of artificial intelligence (Levesque et al., 2012). Taking up this idea author groups Rudinger et al. and Sakaguchi et al. have proposed Winograd based questionnaire framework which can be used to test the presence of gender bias in co-reference resolution systems and word association algorithms respectively (Sakaguchi et al., 2021). In their paper Rudinger et al., the authors present the system with Winograd style sentence-pairs that use profession and differ only in pronouns. Here, the ML system has to predict the pronoun of the profession based on the sentence. For example, the paramedic performed CPR on the passenger even though he/she/they knew it was already too late. The task for the system is to predict the appropriate pronoun for the paramedic (Rudinger et al., 2018).

Similar to Rudinger et al. paper, which detects gender bias in co-reference resolution, the majority of detection methods discussed in these papers target NLP tasks like sentiment analysis (Kiritchenko and Mohammad, 2018; Thelwall, 2018; Sarraf et al., 2021), information retrieval (Rekabsaz and Schedl, 2020), Word Embeddings (Leavy, 2018; Guo and Caliskan, 2021) and a combination of language processing tasks (Hitti et al., 2019; Babaeianjelodar et al., 2020; Dinan et al., 2020) for gender bias detection. Rekabsaz and Schedl have also looked into bias detection methods in Information Retrieval(IR) models. Using metrics like RankBias and AverageRankBias, authors demonstrate that IR models like BERT-Base, BM25, KNRM, MatchPyramid, PACRR, ConvKNRM and BERT-Large are all male inclined (Rekabsaz and Schedl, 2020). The authors use the metrics mentioned above by defining a value, in this case a mathematical representation of the magnitude or occurrences of gender definitive words like *he/him/she/her* in a document and measuring the averages of this value in the rank lists generated by the metrics. While Rekabsaz and Schedl have focused on group fairness the paper by Aggrawal et al. provide a comprehensive fairness detection methods for individual fairness. In this framework they make use of test cases for the algorithm to detect any discriminatory attributes employed by the algorithm to arrive at the prediction (Aggarwal et al., 2019).

Another interesting framework discussed by Li et al. in their paper, is the DENOUNCER (Detection of Unfairness in Classifiers), it is a bias detection framework that takes in training dataset, a set of sensitive attributes in the dataset like race, gender, age etc. and classifiers to be used for the computation. Using these inputs DENOUNCER is able to conduct a fairness detection and present the true vs. the predicted value, hence making it plain for the model creators if their models are biased. For example, if a user wants to check if race is a fair classifier for criminal recidivism prediction. They can use DENOUNCER to select a dataset, COMPAS, and elect a classifier (race of the individual) and run the prediction. The DENOUNCER would run different prediction algorithms and compare the outcome of the prediction (will the individual reoffend) to real outcome (did the individual reoffend) and conduct fairness evaluation of the outcome. Thus, the result will reflect if the classifier selected scored high in fairness evaluation or not (Li et al., 2021). Finally, authors Tramer et al. look into Unwarranted Associate (UA) framework that detects unwanted associations automated systems (Tramer et al., 2017).

### 3.4. Users perspective on gender biases

Out of 120 papers only 8 papers focus on user studies in relation to gender specific biases in ML model. These user studies provide interesting and insightful look into how the ML models are designed, deployed, and perceived. In their paper, Fosch et al. conducted a very short survey across Twitter users to understand and quantify gender-bias present in Twitter's gender assignment algorithm. Twitter like any other social media platform thrives on personal ads that are catered to users based on their race, gender, lifestyle, political leanings and so on. In most cases, when users do not volunteer their gender information Twitter's algorithm assigns a gender to their users inferred from their app activity.

In this study conducted over 4 days with 109 Twitter users, researchers found that for users who did not provide their gender to the platform, the Twitter algorithm misgendered straight men 8% of the time. In contrast the misgendering for gay men and straight women was much higher 25 and 16% respectively. Not surprisingly the non-binary population were misgendered in every case. Furthermore, even if the users tried to update their gender orientation in the platform, the ads were still biased and corresponding to the gender assigned to them. Thus, the only recourse to escape from these ads was to opt out of the personalized ads entirely (Fosch-Villaronga et al., 2021). Although, this was a very short study and the research community needs to conduct more studies like this to get a full picture of the nature of gender bias in the Twitter platform, it is very evident that Twitter's algorithm is significantly discriminatory against non-binary community, and straight women.

A similar study conducted with search engines does a deeper dive into the complex nature of gender bias in both platform and the users of the platform. In their paper, authors Otterbacher et al. use the result of image search results and the Ambivalent Sexism Inventory (ASI) to understand the interaction of gender-bias in the results and user's perception of it. ASI is a scoring system in which participants are measured for two types of sexism: Hostile Sexism (HS) which views minority gender in negative light and Benevolent Sexism (BS) which views minority gender in less negative albeit through stereotypical lenses. In their study, the researchers show users a grid of images and ask the users to guess the query used in the search engine which might have resulted in those images. Then the researchers reveal the actual query used and ask the participants to compare their answer with the query. These questions along with the ASI score helped the researchers to arrive at the conclusion that participants who scored higher in the ASI scale, i.e., displayed sexist tendencies, tended to not see any gender-bias in the biased search result images. This study conducted on 280 participants across US, UK and India reveals that a biased search engine perpetuates gender stereotypes and sexism (Otterbacher et al., 2018), thus further exacerbating the issue.

In their paper, Hitti et al. study user perception of gender bias by conducting a survey on 44 participants. They find that about 90% of the participants understand the concept of gender bias. The participants identify gender stereotypes (100% agreement), Gender Generalization (90% agreement), and abusive language (80% agreement) as three significant sub-types of gender bias (Hitti et al., 2019). Similarly Wang et al. also look into users preference on gender-biased vs. gender-fair systems by conducting an online study on 202 university students. In this study, the researchers also gauge the users perception on their role in perpetuating gender bias in the recommender systems using a career recommender. They find that participants prefer a gender-biased (recommending jobs based on gender stereotypes) system as it confirms with their own implicit biases. Through this study, researchers suggest that gender bias is a societal issue and technical mitigation methods are simply not enough to remove gender biases from automated decision-making systems (Wang and Chen, 2021).

On the other hand, there are also user studies that look into the creation side of gender-biased models. In a unique study, Cowgill et al. study the behavior of close to 400 AI engineers when they are tasked to design a system that predicts standardized test scores for a demographic with a differing circumstance. In this study, the authors cleverly intervene the model creation process with a gender-bias awareness module, to study if such warning or idea changes the creators' model. Here, engineers were asked to study on gender-bias awareness module before continuing their model creation. Surprisingly, after this intervention most of the models tended to over-estimate the test scores for female demographic. This study reveals that bias is a very nuanced topic and more thought should be put into how to educate ML/AI creators on tackling such issue (Cowgill et al., 2020).

Another expert study conducted on ML practitioners reveals the complexity of addressing the issue of bias in application (Chouldechova and Roth, 2020). In their paper, authors Andrus et al. outline different hurdles faced by ML/AI practitioners when they are trying to mitigate gender or racial biases in practice. The majority of practitioner agree that they simply do not have access to demographic data with sensitive information, unless they work in healthcare, employment (HR, recruiting) or financial institution, they cannot gain access to racially balanced datasets. They also talk about the organizational priority and legal limitation that holds them back from vetting their algorithms for biases (Andrus et al., 2021).

### 3.5. Literature reviews

In our study, we found eight papers that also conducted literature reviews on gender-bias in various ML/AI application. Unlike our work, these literature reviews mainly focused on specific algorithmic group or ML/AI implementation for example, NLP. In their paper, Khalil et al. review 24 academic publications to analyze the gender-bias in Facial Analysis Technology. Through their literature review the researchers show that facial analysis systems rely heavily on stereotypes to classify ambiguous facial features, thus leading to gender biases. The authors pull readers focus into the importance of algorithmic auditing and more academic research. Authors argue that by providing more attention into this issue, we can invoke positive action to prevent and mitigate gender biases in image classification (Khalil et al., 2020). An interesting paper by O'Reilly Shah also looks into the gender bias in the field of medicine (O'Reilly-Shah et al., 2020).

In their paper, Blodgett et al. review 146 papers published on gender-bias analysis in the NLP systems. In this paper, the authors recommend implementing proper data vetting and understanding the context of social norms and language use to proactively mitigate biases in NLP systems. Through their paper, authors present the solution of fixing the gender-bias problem from outside-in, i.e., proactively understanding the context and data rather than fixing the algorithm after training (Blodgett et al., 2020). Sun et al. also conducted literature review on the presence of gender-bias in NLP systems. However, unlike the other papers, this paper focuses on the mitigation methods presented by the fellow authors to prevent gender-biases (Sun et al., 2019). We also found other literature review papers that focus on gender biases in academic literature (Bird et al., 2019; Cramer et al., 2019; Boratto et al., 2021; Savoldi et al., 2021).

### 3.6. Case studies

Our data search resulted in five papers focused on case studies demonstrating gender biases in automated decision-making systems. Case studies are instrumental because they display the inner mechanism of implementing these opaque systems and illuminate essential details specific to that system. Thus, allowing readers to understand and follow the process of decision-making adopted by these automated systems.

For example, the paper by Prates et al. provides a detailed case study into Google Translate. Google Translate is a powerful machine translation tool that is within reach of many people. Due to its ease of access, cost-free use, and popularity (200 million users daily), it is imperative to understand if this machine translation tool is gender biased. The authors conducted a quantitative analysis of Google Translate using gender-neutral languages supported by the system, which included 14 languages including Malay, Estonian, Finnish, Hungarian, Bengali, Swahili, Chinese, and others. Using statistical translation tools, they show that Google Translate is gender-biased toward male defaults (tends to default to he/him/his pronouns more frequently) without any reason. It assigns she/her/hers pronouns when adjectives like *Shy* or *Desirable* are used, and it overwhelmingly assigns he/him/his pronouns when STEM profession words are used (Prates et al., 2020). Farkas and Nemeth extend this study with Hungarian labor data and found that occupation-related words tend to be more biased than adjectives (Farkas and Németh, 2022).

Another case study looks into the Automated Deception Detection tool and tries to see if the prediction provided by the software is stereotyping non-verbal behaviors (NVB) cues given by people. Crockett et al. utilize raw video data collected from 32 participants to test if there is a statistically significant gender effect on the deception detection system. Through this, they find a gender effect in NVB cues generated by people, which means we cannot use a system trained on female data to detect deception on male participants and vice-versa (Crockett et al., 2020). The paper by Wang et al. also conducts a case study into career recommender systems and implementation of debiasing technique (Wang et al., 2021). Finally, in their paper, Dutta et al. display the effect of debiasing techniques like feature hashing on the performance of automated classification systems. Although the debiasing technique resulted in a fairer system as measured by the Difference of Equal Odds metric, it causes a drop of 6.1% in the overall accuracy of the classification (Dutta et al., 2020).

## 4. Discussion and implication

Majority of the papers, except the literature reviews, have mainly focused on one specific ML/AI model or model-family, like language processing, image processing or recommender systems and so on. Although, the mitigation and detection technique discussed in these papers can be extended to other ML models as well, the specificity of these measures shows how far-reaching and nuanced ML/AI applications are. Furthermore, we have identified following topic of interest that could benefit from more future research.

### 4.1. What is gender bias

Most papers reviewed in our study provide valuable insights into the presence of gender bias and, practical bias mitigation and detection methods. While these insights are important, most papers do not address what constitutes gender bias. As the ML/AI assisted systems are increasingly proliferating society, these biased systems will have direct impact on users who are unaware of these biases. Also, because of the nature of gender bias and its presence in our society and our implicit choices it is tricky for users themselves to identify a biased automated system. It is difficult to hold systems accountable for their unfair treatment when users are unable to understand what unfair treatments look like. As pointed out by researchers like Otterbacher et al., unchecked gender biases in automated systems in combination with users' implicit biases can create a regressive feedback loop that pose risk to the gender fairness movement overall (Otterbacher et al., 2018).

Thus, the research should outline how gender biases manifests in automated decision-making systems in its varied applications. Papers by researchers like Melchiorre et al. point out that the prediction accuracy for minority population drops in the biased system (Melchiorre et al., 2021). However, technical terms like prediction accuracy might not be easy to understand and communicate in many users. Moreover, providing clear definition and identification of a gender-biased system can assist in effective policing and monitoring of ML/AI assisted systems that directly impacts users.

### 4.2. Algorithmic accountability

Currently, there is a lack of a legal framework that oversees the design and development of the automated decision-making systems. This lack of rules has allowed companies and organizations to overlook their part in the gender-bias issue. As pointed out in the research by Srinivas et al. many off-the-shelf and government prescribed systems have gender biases even when the model designers claim that they have created a fair system. There are no clear legal repercussions for creating an unfair system or for making false claims (Srinivas et al., 2019).

Even when the model designers and creators want to take steps in vetting the data or implementing mitigation efforts, they do not have enough resources, access, or backing from either the companies or the government. As revealed by professionals in the field (Chouldechova and Roth, 2020), due to these limitations and corporate agendas taking precedence, there is no improvement in the gender-bias issue even when there are multiple solutions available. Hence, we need more research into the lack of government action and legal slump regarding gender-bias issues to push the issues further. We also need to explore the hurdles, legal and ethical dilemma faced by algorithm designers who have limited or no access to comprehensive and representative data to train a fair model.

### 4.3. Interdisciplinary approach

The papers reviewed in the study show that gender-bias issues are present in all applications of automated-decision-making systems. These systems pose risks to users belonging to minority gender groups. As pointed out by several researchers in their papers, field knowledge is very important when creating an effective and just system. In order to understand the extent of damage and remedy, we need support from experts in these diverse fields. Their field knowledge and insight can guide the automated-systems designers and researchers to spot the risk factor and lend support to the remediation of these issues. For example, if a social program is utilizing the automated decision- support system then an expert within in the field of social work and policymaking should also be involved in the process of selecting/designing and vetting the decision-support system that will be implemented.

### 4.4. Missing user perception

Through this study, we have discovered that the user perception of these ML/AI application is largely unexplored. Users play a vital role in bias recognition and success of mitigation methods. As pointed out by several papers in this study, there is great digital divide between the creators of ML/AI systems and the users who benefit from these systems. User likeability and trust into ML/AI assisted decision-making system is equally or more important than the functionality and efficiency of the system for the successful integration. As we have discovered in this study, there were only 8 papers that leveraged user studies to understand how users perceive, comprehend, and utilize these systems.

In our study, user studies have demonstrated the implicit bias existing on users (Wang and Chen, 2021) and how these biases can exacerbate the gender bias further (Otterbacher et al., 2018). Additionally, user studies like the one conducted by Andrus et al. on ML/AI practitioners, also shed light on the ground-reality of model creators who are trying to create a fair system but are unable to do so due to various organizational and legal hurdles (Andrus et al., 2021).

Considering the rapid pace at which these systems are being implemented into our lives, 8 user studies is very low. Thus, it is even more important to include user views and experiences with these applications into the larger discussion of gender biases in ML systems. Especially in the context of gender fairness, we need to conduct more studies to understand the experiences of non-binary population with these systems, as their representation is largely missing from both the algorithm design process and data used in model training.

Finally, there is no denying that automated systems provide immense advantages to us and push us forward into modern civilization. Automated systems lend us the capability to actualize the fourth industrial revolution. However, as contributors of technology society we need to be cognizant of the fact that these systems might have varied effect of population of different social strata. It is our social duty and responsibility to bring everyone along into the fold into the new age of innovation. The progress of automated systems depends on majority of population being able to understand and trust these systems. A lack of understanding and trust will result into delays and conflicts within the society. Hence, it is imperative that we strive to create a fair automated system that benefits all users.

## 5. Limitation and future work

Systematic literature review is one of the most useful methods to learn about the research trends in any given domain, but it also leaves the possibility of some limitations. During our review process, we have done our due diligence to obtain all the relevant papers, however, it is likely that due to technical limitations (like lack of time, not using other possible keywords for search, not utilizing other database search platforms) we might have missed some papers which addressed and analyzed the impact of machine learning biases. We aim to address our limitation in the future by looking into more literature published in recent times and in different languages to get a better view of the current research landscape focusing on gender bias in automated decision-making systems. We also aim to conduct case studies and user studies to further extend our work in gender bias in ML/AI systems.

## 6. Conclusion

ML/AI assisted systems have seamlessly integrated into our lives, quietly manipulating the items we buy, the entertainment we see and the doctors we visit. In our study, we reviewed n = 120 academic literature published on gender bias in automated decision-making systems. Through the literature review we identify the different areas explored in the research that have demonstrated presence of gender bias. We review different methods of study utilized in the papers to understand and analyze the state of gender bias in automated systems. We also detail the bias detection and mitigation methods proposed by the researchers. Finally, we highlight the areas that require more focus in the future research to further push the conversation of gender bias in ML/AI assisted systems. In conclusion, we believe the research into gender-bias in ML/AI assisted system should provide the definition and identification of a gender-biased system. Also, researchers should promote algorithmic auditing and interdisciplinary approach to design and develop ML/AI systems. Researchers should also conduct more user studies to bring the users perception into a biased system.

## Data availability statement

The original contributions presented in the study are included in the article/supplementary material, further inquiries can be directed to the corresponding author/s.

## Author contributions

SS has collected the papers analyzed and conducted the analysis as well as reporting of the data. SD has worked on the study ideation, analysis, design, and parts of writing the work. Both authors contributed to the article and approved the submitted version.

## Conflict of interest

The authors declare that the research was conducted in the absence of any commercial or financial relationships that could be construed as a potential conflict of interest.

## Publisher's note

All claims expressed in this article are solely those of the authors and do not necessarily represent those of their affiliated organizations, or those of the publisher, the editors and the reviewers. Any product that may be evaluated in this article, or claim that may be made by its manufacturer, is not guaranteed or endorsed by the publisher.

## Author disclaimer

Any opinions, findings, and conclusions or recommendations expressed in this material are solely those of the authors and do not necessarily reflect the views of the University of Denver.

## Footnotes

^1^ https://aif360.readthedocs.io/en/latest/modules/generated/aif360.sklearn.inprocessing.AdversarialDebiasing.html

## References

[B1] Aggarwal A. Lohia P. Nagar S. Dey K. Saha D. (2019). “Black box fairness testing of machine learning models,” in Proceedings of the 2019 27th ACM Joint Meeting on European Software Engineering Conference and Symposium on the Foundations of Software Engineering, ESEC/FSE 2019 (New York, NY: Association for Computing Machinery), 625–635. 10.1145/3338906.3338937

[B2] Amershi S. Weld D. Vorvoreanu M. Fourney A. Nushi B. Collisson P. . (2019). “Guidelines for human-AI interaction,” in Proceedings of the 2019 CHI Conference on Human Factors in Computing Systems (New York, NY), 1–13. 10.1145/3290605.3300233

[B3] Andrus M. Spitzer E. Brown J. Xiang A. (2021). “What we can't measure, we can't understand: challenges to demographic data procurement in the pursuit of fairness,” in Proceedings of the 2021 ACM Conference on Fairness, Accountability, and Transparency (New York, NY), 249–260. 10.1145/3442188.3445888

[B4] Babaeianjelodar M. Lorenz S. Gordon J. Matthews J. Freitag E. (2020). “Quantifying gender bias in different corpora,” in Companion Proceedings of the Web Conference 2020 (New York, NY), 752–759. 10.1145/3366424.3383559

[B5] Baeza-Yates R. (2020). Bias in Search and Recommender Systems. New York, NY: Association for Computing Machinery. 10.1145/3383313.3418435

[B6] Balakrishnan G. Xiong Y. Xia W. Perona P. (2021). Towards Causal Benchmarking of Biasin Face Analysis Algorithms. Cham: Springer International Publishing. 10.1007/978-3-030-74697-1_15

[B7] Barnabó G. Fazzone A. Leonardi S. Schwiegelshohn C. (2019). “Algorithms for fair team formation in online labour marketplaces,” in Companion Proceedings of The 2019 World Wide Web Conference (New York, NY), 484–490. 10.1145/3308560.3317587

[B8] Bender E. M. Friedman B. (2018). Data statements for natural language processing: toward mitigating system bias and enabling better science. Trans. Assoc. Comput. Linguist. 6, 587–604. 10.1162/tacl_a_00041

[B9] Bird S. Kenthapadi K. Kiciman E. Mitchell M. (2019). “Fairness-aware machine learning: practical challenges and lessons learned,” in Proceedings of the Twelfth ACM International Conference on Web Search and Data Mining, WSDM '19 (New York, NY: Association for Computing Machinery), 834–835. 10.1145/3289600.3291383

[B10] Blodgett S. L. Barocas S. Daumé H. III. Wallach H. (2020). Language (technology) is power: a critical survey of “bias” in NLP. arXiv preprint arXiv:2005.14050. 10.18653/v1/2020.acl-main.485

[B11] Booth B. M. Hickman L. Subburaj S. K. Tay L. Woo S. E. D'Mello S. K. (2021). “Bias and fairness in multimodal machine learning: a case study of automated video interviews,” in Proceedings of the 2021 International Conference on Multimodal Interaction, 268–277. 10.1145/3462244.3479897

[B12] Boratto L. Faralli S. Marras M. Stilo G. (2021). Report on the international workshop on algorithmic bias in search and recommendation (bias 2020). SIGIR Forum 54, 1–5. 10.1145/3451964.3451973

[B13] Brandao M. (2019). Age and gender bias in pedestrian detection algorithms. arXiv preprint arXiv:1906.10490. 10.48550/ARXIV.1906.10490

[B14] Buolamwini J. Gebru T. (2018). “Gender shades: intersectional accuracy disparities in commercial gender classification,” in Proceedings of the 1st Conference on Fairness, Accountability and Transparency, eds S. A. Friedler and C. Wilson, 77–91.

[B15] Caton S. Haas C. (2020). Fairness in machine learning: a survey. arXiv preprint arXiv:2010.04053. 10.48550/ARXIV.2010.04053

[B16] Chakraborty J. Majumder S. Yu Z. Menzies T. (2020). “Fairway: a way to build fair ML software,” in Proceedings of the 28th ACM Joint Meeting on European Software Engineering Conference and Symposium on the Foundations of Software Engineering (New York, NY), 654–665. 10.1145/3368089.3409697

[B17] Chen Y. Mahoney C. Grasso I. Wali E. Matthews A. Middleton T. . (2021). Gender Bias and Under-Representation in Natural Language Processing Across Human Languages. New York, NY: Association for Computing Machinery. 10.1145/3461702.3462530

[B18] Cho W. I. Kim J. Yang J. Kim N. S. (2021). “Towards cross-lingual generalization of translation gender bias,” in Proceedings of the 2021 ACM Conference on Fairness, Accountability, and Transparency (New York, NY), 449–457. 10.1145/3442188.3445907

[B19] Chouldechova A. Roth A. (2020). A snapshot of the frontiers of fairness in machine learning. Commun. ACM 63, 82–89. 10.1145/3376898

[B20] Courtland R. (2018). The bias detectives. Nature 558, 357–360. 10.1038/d41586-018-05469-3 29925973

[B21] Cowgill B. Dell'Acqua F. Deng S. Hsu D. Verma N. Chaintreau A. (2020). “Biased programmers? or biased data? a field experiment in operationalizing AI ethics,” in Proceedings of the 21st ACM Conference on Economics and Computation (New York, NY), 679–681. 10.1145/3391403.3399545

[B22] Cramer H. Garcia-Gathright J. Reddy S. Springer A. Takeo Bouyer R. (2019). “Translation, tracks & data: an algorithmic bias effort in practice,” in Extended Abstracts of the 2019 CHI Conference on Human Factors in Computing Systems, CHI EA '19 (New York, NY: Association for Computing Machinery), 1–8.

[B23] Cramer H. Garcia-Gathright J. Springer A. Reddy S. (2018). Assessing and addressing algorithmic bias in practice. Interactions 25, 58–63. 10.1145/3278156

[B24] Crockett K. O'Shea J. Khan W. (2020). “Automated deception detection of males and females from non-verbal facial micro-gestures,” in 2020 International Joint Conference on Neural Networks (IJCNN) (Glasgow), 1–7. 10.1109/IJCNN48605.2020.9207684

[B25] Das A. Dantcheva A. Bremond F. (2018). “Mitigating bias in gender, age and ethnicity classification: a multi-task convolution neural network approach,” in Proceedings of the European Conference on Computer Vision (ECCV) workshops (Munich). 10.1007/978-3-030-11009-3_35

[B26] Das S. Wang B. Tingle Z. Camp L. J. (2019). “Evaluating user perception of multi-factor authentication: a systematic review,” in Proceedings of the Thriteenth International Symposium on Human Aspects of Information Security & Assurance (HAISA 2019) (Nicosia).

[B27] Dass R. K. Petersen N. Visser U. Omori M. (2020). “It's not just black and white: classifying defendant mugshots based on the multidimensionality of race and ethnicity,” in 2020 17th Conference on Computer and Robot Vision (CRV) (IEEE), 238–245. 10.1109/CRV50864.2020.00039

[B28] De-Arteaga M. Romanov A. Wallach H. Chayes J. Borgs C. Chouldechova A. . (2019). “Bias in bios: a case study of semantic representation bias in a high-stakes setting,” in Proceedings of the Conference on Fairness, Accountability, and Transparency, FAT '19 (New York, NY: Association for Computing Machinery), 120–128. 10.1145/3287560.3287572

[B29] Dhar P. Gleason J. Souri H. Castillo C. D. Chellappa R. (2020). Towards gender-neutral face descriptors for mitigating bias in face recognition. arXiv preprint arXiv:2006.07845. 10.48550/ARXIV.2006.07845

[B30] D'Ignazio C. Graeff E. Harrington C. N. Rosner D. K. (2020). Toward Equitable Participatory Design: Data Feminism for CSCW amidst Multiple Pandemics. New York, NY: Association for Computing Machinery. 10.1145/3406865.3418588

[B31] Dinan E. Fan A. Wu L. Weston J. Kiela D. Williams A. (2020). Multi-dimensional gender bias classification. arXiv preprint arXiv:2005.00614. 10.18653/v1/2020.emnlp-main.23

[B32] Donnelly N. Stapleton L. (2021). Digital enterprise technologies: do enterprise control and automation technologies reinforce gender biases and marginalisation? IFAC Pap. Online 54, 551–556. 10.1016/j.ifacol.2021.10.507

[B33] Dutta R. Gohil V. Jain A. (2020). “Effect of feature hashing on fair classification,” in Proceedings of the 7th ACM IKDD CoDS and 25th COMAD, CoDS COMAD 2020 (New York, NY: Association for Computing Machinery), 365–366. 10.1145/3371158.3371230

[B34] Farkas A. Németh R. (2022). How to measure gender bias in machine translation: real-world oriented machine translators, multiple reference points. Soc. Sci. Human. Open 5, 100239. 10.1016/j.ssaho.2021.100239

[B35] Farnad G. Babaki B. Gendreau M. (2020). “A unifying framework for fairness-aware influence maximization,” in Companion Proceedings of the Web Conference 2020 (New York, NY), 714–722. 10.1145/3366424.3383555

[B36] Feldman T. Peake A. (2021). End-to-end bias mitigation: removing gender bias in deep learning. arXiv [Preprint]. arXiv: 2104.02532. 10.48550/ARXIV.2104.02532

[B37] Fleisher W. (2021). “What's fair about individual fairness?” in Proceedings of the 2021 AAAI/ACM Conference on AI, Ethics, and Society (New York, NY), 480–490. 10.1145/3461702.3462621

[B38] Folkerts F. Schreck V. Riazy S. Simbeck K. (2019). “Analyzing sentiments of German job references,” in 2019 IEEE International Conference on Humanized Computing and Communication (HCC) (Laguna Hills, CA: IEEE), 1–6. 10.1109/HCC46620.2019.00009

[B39] Fosch-Villaronga E. Poulsen A. Søraa R. A. Custers B. (2021). Gendering algorithms in social media. ACM SIGKDD Explorat. Newsletter 23, 24–31. 10.1145/3468507.3468512

[B40] Geyik S. C. Ambler S. Kenthapadi K. (2019). “Fairness-aware ranking in search & recommendation systems with application to linkedin talent search,” in Proceedings of the 25th ACM SIGKDD International Conference on Knowledge Discovery & *Data Mining*, 2221–2231. 10.1145/3292500.3330691

[B41] Gilbert T. K. Mintz Y. (2019). “Epistemic therapy for bias in automated decision-making,” in Proceedings of the 2019 AAAI/ACM Conference on AI, Ethics, and Society, 61–67. 10.1145/3306618.3314294

[B42] Glymour B. Herington J. (2019). “Measuring the biases that matter: the ethical and casual foundations for measures of fairness in algorithms,” in Proceedings of the Conference on Fairness, Accountability, and Transparency, 269–278. 10.1145/3287560.3287573

[B43] Guo W. Caliskan A. (2021). Detecting Emergent Intersectional Biases: Contextualized Word Embeddings Contain a Distribution of Human-like Biases. New York, NY: Association for Computing Machinery. 10.1145/3461702.3462536

[B44] Gutierrez M. (2021). New feminist studies in audiovisual industries| algorithmic gender bias and audiovisual data: a research agenda. Int. J. Commun. 15, 23.

[B45] Hicks M. (2019). Hacking the Cis-TEM. IEEE Ann. History Comput. 41, 20–33. 10.1109/MAHC.2019.2897667

[B46] Hitti Y. Jang E. Moreno I. Pelletier C. (2019). “Proposed taxonomy for gender bias in text; a filtering methodology for the gender generalization subtype,” in Proceedings of the First Workshop on Gender Bias in Natural Language Processing (Florence: Association for Computational Linguistics), 8–17. 10.18653/v1/W19-3802

[B47] Hong J. Zhu Z. Yu S. Wang Z. Dodge H. H. Zhou J. (2021). “Federated adversarial debiasing for fair and transferable representations,” in Proceedings of the 27th ACM SIGKDD Conference on Knowledge Discovery & Data Mining, 617–627. 10.1145/3447548.3467281 35571559 PMC9105979

[B48] Howard A. Borenstein J. (2018). The ugly truth about ourselves and our robot creations: the problem of bias and social inequity. Sci. Eng. Ethics 24, 1521–1536. 10.1007/s11948-017-9975-2 28936795

[B49] Howcroft D. Rubery J. (2019). “Bias in, bias out”: gender equality and the future of work debate. Labour Industry 29, 213–227. 10.1080/10301763.2019.1619986

[B50] Jia S. Meng T. Zhao J. Chang K.-W. (2020). Mitigating gender bias amplification in distribution by posterior regularization. arXiv preprint arXiv:2005.06251. 10.18653/v1/2020.acl-main.264

[B51] Karimi-Haghighi M. Castillo C. (2021). “Enhancing a recidivism prediction tool with machine learning: effectiveness and algorithmic fairness,” in Proceedings of the Eighteenth International Conference on Artificial Intelligence and Law, 210–214. 10.1145/3462757.3466150

[B52] Kärkkäinen K. Joo J. (2019). Fairface: face attribute dataset for balanced race, gender, and age. arXiv preprint arXiv:1908.04913. 10.48550/ARXIV.1908.04913

[B53] Katell M. Young M. Dailey D. Herman B. Guetler V. Tam A. . (2020). “Toward situated interventions for algorithmic equity: lessons from the field,” in Proceedings of the 2020 Conference on Fairness, Accountability, and Transparency, 45–55. 10.1145/3351095.3372874

[B54] Khalil A. Ahmed S. G. Khattak A. M. Al-Qirim N. (2020). Investigating bias in facial analysis systems: a systematic review. IEEE Access 8, 130751–130761. 10.1109/ACCESS.2020.3006051

[B55] Kiritchenko S. Mohammad S. M. (2018). Examining gender and race bias in two hundred sentiment analysis systems. arXiv preprint arXiv:1805.04508. 10.18653/v1/S18-2005

[B56] Koene A. Dowthwaite L. Seth S. (2018). “IEEE P7003TM standard for algorithmic bias considerations: work in progress paper,” in Proceedings of the International Workshop on Software Fairness, 38–41. 10.1145/3194770.3194773

[B57] Krishnan A. Almadan A. Rattani A. (2020). “Understanding fairness of gender classification algorithms across gender-race groups,” in 2020 19th IEEE International Conference on Machine Learning and Applications (ICMLA) (IEEE), 1028–1035. 10.1109/ICMLA51294.2020.00167

[B58] Krüger S. Hermann B. (2019). “Can an online service predict gender? on the state-of-the-art in gender identification from texts,” in 2019 IEEE/ACM 2nd International Workshop on Gender Equality in Software Engineering (GE) (Los Alamitos, CA: IEEE), 13–16. 10.1109/GE.2019.00012

[B59] Lambrecht A. Tucker C. (2019). Algorithmic bias? An empirical study of apparent gender-based discrimination in the display of stem career ads. Manage. Sci. 65, 2966–2981. 10.1287/mnsc.2018.3093

[B60] Leavy S. (2018). “Gender bias in artificial intelligence: the need for diversity and gender theory in machine learning,” in Proceedings of the 1st International Workshop on Gender Equality in Software Engineering, GE '18 (New York, NY: Association for Computing Machinery), 14–16. 10.1145/3195570.3195580

[B61] Levesque H. Davis E. Morgenstern L. (2012). “The winograd schema challenge,” in Thirteenth International Conference on the Principles of Knowledge Representation and Reasoning (Rome).

[B62] Li J. Moskovitch Y. Jagadish H. V. (2021). Denouncer: detection of unfairness in classifiers. Proc. VLDB Endow. 14, 2719–2722. 10.14778/3476311.3476328

[B63] Liu H. Wang W. Wang Y. Liu H. Liu Z. Tang J. (2020). Mitigating gender bias for neural dialogue generation with adversarial learning. arXiv preprint arXiv:2009.13028. 10.18653/v1/2020.emnlp-main.64

[B64] Lopez S. Yang Y. Beltran K. Kim S. J. Cruz Hernandez J. Simran C. . (2019). “Investigating implicit gender bias and embodiment of white males in virtual reality with full body visuomotor synchrony,” in Proceedings of the 2019 CHI Conference on Human Factors in Computing Systems, CHI '19 (New York, NY: Association for Computing Machinery). 10.1145/3290605.3300787

[B65] Lu K. Mardziel P. Wu F. Amancharia P. Datta A. (2020). “Gender bias in neural natural language processing,” in Logic, Language, and Security: Essays Dedicated to Andre Scedrov on the Occasion of His 65th Birthday (Cham: Springer), 189–202. 10.48550/ARXIV.1807.11714

[B66] Manresa-Yee C. Ramis S. (2021). “Assessing gender bias in predictive algorithms using explainable AI,” in Proceedings of the XXI International Conference on Human Computer Interaction, 1–8. 10.1145/3471391.3471420

[B67] Maudslay R. H. Gonen H. Cotterell R. Teufel S. (2019). It's all in the name: mitigating gender bias with name-based counterfactual data substitution. arXiv preprint arXiv:1909.00871. 10.48550/ARXIV.1909.00871

[B68] Mehrabi N. Morstatter F. Saxena N. Lerman K. Galstyan A. (2021). A survey on bias and fairness in machine learning. ACM Comput. Surv. 54, 1–35. 10.1145/3457607

[B69] Melchiorre A. B. Rekabsaz N. Parada-Cabaleiro E. Brandl S. Lesota O. Schedl M. (2021). Investigating gender fairness of recommendation algorithms in the music domain. Inform. Process. Manage. 58, 102666. 10.1016/j.ipm.2021.102666

[B70] Mishra A. Mishra H. Rathee S. (2019). Examining the presence of gender bias in customer reviews using word embedding. arXiv preprint arXiv:1902.00496. 10.2139/ssrn.3327404

[B71] Molina D. A. Causa L. Tapia J. (2020). “Reduction of bias for gender and ethnicity from face images using automated skin tone classification,” in 2020 International Conference of the Biometrics Special Interest Group (BIOSIG) (IEEE), 1–5.

[B72] Morales A. Fierrez J. Vera-Rodriguez R. Tolosana R. (2020). Sensitivenets: learning agnostic representations with application to face images. IEEE Trans. Pattern Anal. Mach. Intell. 43, 2158–2164. 10.1109/TPAMI.2020.3015420 32776875

[B73] Narla A. Kuprel B. Sarin K. Novoa R. Ko J. (2018). Automated classification of skin lesions: from pixels to practice. J. Investig. Dermatol. 138, 2108–2110. 10.1016/j.jid.2018.06.175 30244720

[B74] Novin A. Meyers E. (2017). “Making sense of conflicting science information: exploring bias in the search engine result page,” in Proceedings of the 2017 Conference on Conference Human Information Interaction and Retrieval, 175–184. 10.1145/3020165.3020185

[B75] O'Reilly-Shah V. N. Gentry K. R. Walters A. M. Zivot J. Anderson C. T. Tighe P. J. (2020). Bias and ethical considerations in machine learning and the automation of perioperative risk assessment. Brit. J. Anaesthesia 125, 843–846. 10.1016/j.bja.2020.07.040 32838979 PMC7442146

[B76] Otterbacher J. Checco A. Demartini G. Clough P. (2018). “Investigating user perception of gender bias in image search: the role of sexism,” in The 41st International ACM SIGIR Conference on Research & Development in Information Retrieval, 933–936. 10.1145/3209978.3210094

[B77] Paviglianiti A. Pasero E. (2020). “Vital-ECG: a de-bias algorithm embedded in a gender-immune device,” in 2020 IEEE International Workshop on Metrology for Industry 4.0 & IoT (IEEE), 314–318. 10.1109/MetroInd4.0IoT48571.2020.9138291

[B78] Pena A. Serna I. Morales A. Fierrez J. (2020). “Bias in multimodal AI: testbed for fair automatic recruitment,” in 2020 IEEE/CVF Conference on Computer Vision and Pattern Recognition Workshops (CVPRW), 129–137. 10.1109/CVPRW50498.2020.00022

[B79] Prates M. O. Avelar P. H. Lamb L. C. (2020). Assessing gender bias in machine translation: a case study with google translate. Neural Comput. Appl. 32, 6363–6381. 10.1007/s00521-019-04144-6

[B80] Prost F. Thain N. Bolukbasi T. (2019). Debiasing embeddings for reduced gender bias in text classification. arXiv preprint arXiv:1908.02810. 10.18653/v1/W19-3810

[B81] Raghavan M. Barocas S. Kleinberg J. Levy K. (2020). “Mitigating bias in algorithmic hiring: evaluating claims and practices,” in Proceedings of the 2020 Conference on Fairness, Accountability, and Transparency (New York, NY), 469–481. 10.1145/3351095.3372828

[B82] Raz D. Bintz C. Guetler V. Tam A. Katell M. Dailey D. . (2021). Face Mis-ID: An Interactive Pedagogical Tool Demonstrating Disparate Accuracy Rates in Facial Recognition. New York, NY: Association for Computing Machinery. 10.1145/3461702.3462627

[B83] Rekabsaz N. Schedl M. (2020). “Do neural ranking models intensify gender bias?” in Proceedings of the 43rd International ACM SIGIR Conference on Research and Development in Information Retrieval, 2065–2068. 10.1145/3397271.3401280

[B84] Righetti L. Madhavan R. Chatila R. (2019). Unintended consequences of biased robotic and artificial intelligence systems [ethical, legal, and societal issues]. IEEE Robot. Automat. Mag. 26, 11–13. 10.1109/MRA.2019.2926996

[B85] Rudinger R. Naradowsky J. Leonard B. Van Durme B. (2018). Gender bias in coreference resolution. arXiv preprint arXiv:1804.09301. 10.18653/v1/N18-2002

[B86] Sakaguchi K. Bras R. L. Bhagavatula C. Choi Y. (2021). Winogrande: an adversarial winograd schema challenge at scale. Commun. ACM 64, 99–106. 10.1145/3474381

[B87] Santana B. S. Woloszyn V. Wives L. K. (2018). Is there gender bias and stereotype in portuguese word embeddings? arXiv preprint arXiv:1810.04528. 10.48550/ARXIV.1810.04528

[B88] Sarraf D. Vasiliu V. Imberman B. Lindeman B. (2021). Use of artificial intelligence for gender bias analysis in letters of recommendation for general surgery residency candidates. Am. J. Surg. 222, 1051–1059. 10.1016/j.amjsurg.2021.09.034 34674847

[B89] Savoldi B. Gaido M. Bentivogli L. Negri M. Turchi M. (2021). Gender bias in machine translation. Trans. Assoc. Comput. Linguist. 9, 845–874. 10.1162/tacl_a_00401

[B90] Schwemmer C. Knight C. Bello-Pardo E. D. Oklobdzija S. Schoonvelde M. Lockhart J. W. (2020). Diagnosing gender bias in image recognition systems. Socius 6, 2378023120967171. 10.1177/2378023120967171 35936509 PMC9351609

[B91] Serna I. Pena A. Morales A. Fierrez J. (2021). “Insidebias: measuring bias in deep networks and application to face gender biometrics,” in 2020 25th International Conference on Pattern Recognition (ICPR) (IEEE), 3720–3727. 10.1109/ICPR48806.2021.9412443

[B92] Shakespeare D. Porcaro L. Gómez E. Castillo C. (2020). Exploring artist gender bias in music recommendation. arXiv preprint arXiv:2009.01715. 10.48550/ARXIV.2009.01715

[B93] Shein E. (2018). The dangers of automating social programs. Commun. ACM 61, 17–19. 10.1145/3264627

[B94] Shekhawat N. Chauhan A. Muthiah S. B. (2019). “Algorithmic privacy and gender bias issues in google ad settings,” in Proceedings of the 10th ACM Conference on Web Science, 281–285. 10.1145/3292522.3326033

[B95] Singh V. K. Chayko M. Inamdar R. Floegel D. (2020). Female librarians and male computer programmers? Gender bias in occupational images on digital media platforms. J. Assoc. Inform. Sci. Technol. 71, 1281–1294. 10.1002/asi.24335

[B96] Singh V. K. Hofenbitzer C. (2019). “Fairness across network positions in cyberbullying detection algorithms,” in 2019 IEEE/ACM International Conference on Advances in Social Networks Analysis and Mining (ASONAM) (IEEE), 557–559. 10.1145/3341161.3342949

[B97] Smith P. Ricanek K. (2020). “Mitigating algorithmic bias: evolving an augmentation policy that is non-biasing,” in Proceedings of the IEEE/CVF Winter Conference on Applications of Computer Vision Workshops (Snowmass, CO), 90–97. 10.1109/WACVW50321.2020.9096905

[B98] Srinivas N. Hivner M. Gay K. Atwal H. King M. Ricanek K. (2019). “Exploring automatic face recognition on match performance and gender bias for children,” in 2019 IEEE Winter Applications of Computer Vision Workshops (WACVW) (Waikoloa, HI), 107–115. 10.1109/WACVW.2019.00023

[B99] Stanovsky G. Smith N. A. Zettlemoyer L. (2019). Evaluating gender bias in machine translation. arXiv preprint arXiv:1906.00591. 10.18653/v1/P19-1164

[B100] Stowell E. Lyson M. C. Saksono H. Wurth R. C. Jimison H. Pavel M. . (2018). Designing and evaluating mhealth interventions for vulnerable populations: a systematic review,” in Proceedings of the 2018 CHI Conference on Human Factors in Computing Systems, 1–17. 10.1145/3173574.3173589

[B101] Sun T. Gaut A. Tang S. Huang Y. ElSherief M. Zhao J. . (2019). Mitigating gender bias in natural language processing: literature review. arXiv preprint arXiv:1906.08976. 10.18653/v1/P19-1159

[B102] Tang S. Zhang X. Cryan J. Metzger M. J. Zheng H. Zhao B. Y. (2017). Gender bias in the job market: a longitudinal analysis. Proc. ACM Hum. Comput. Interact. 1, 1–19. 10.1145/3134734

[B103] Thelwall M. (2018). Gender bias in sentiment analysis. Online Inform. Rev. 42, 45–57 10.1108/OIR-05-2017-0139

[B104] Tramer F. Atlidakis V. Geambasu R. Hsu D. Hubaux J.-P. Humbert M. . (2017). “Fairtest: discovering unwarranted associations in data-driven applications,” in 2017 IEEE European Symposium on Security and Privacy (EuroS&P) (Paris: IEEE), 401–416. 10.1109/EuroSP.2017.29

[B105] Vasudevan S. Kenthapadi K. (2020). “Lift: a scalable framework for measuring fairness in ML applications,” in Proceedings of the 29th ACM International Conference on Information & Knowledge Management (New York, NY), 2773–2780. 10.1145/3340531.3412705

[B106] Wang C. Wang K. Bian A. Islam R. Keya K. N. Foulde J. . (2021). Bias: Friend or Foe? User Acceptance of Gender Stereotypes in Automated Career Recommendations. UMBC Student Collection.

[B107] Wang N. Chen L. (2021). “User bias in beyond-accuracy measurement of recommendation algorithms,” in Fifteenth ACM Conference on Recommender Systems (New York, NY), 133–142. 10.1145/3460231.3474244

[B108] Wang T. Lin X. V. Rajani N. F. McCann B. Ordonez V. Xiong C. (2020). Double-hard debias: tailoring word embeddings for gender bias mitigation. arXiv preprint arXiv:2005.00965. 10.18653/v1/2020.acl-main.484

[B109] Wang T. Zhao J. Yatskar M. Chang K.-W. Ordonez V. (2019). “Balanced datasets are not enough: Estimating and mitigating gender bias in deep image representations,” in Proceedings of the IEEE/CVF International Conference on Computer Vision, 5310–5319. 10.1109/ICCV.2019.00541

[B110] Wang Z. Hale S. Adelani D. I. Grabowicz P. Hartman T. Flöck F. . (2019). “Demographic inference and representative population estimates from multilingual social media data,” in The World Wide Web Conference, WWW '19 (New York, NY: Association for Computing Machinery), 2056–2067. 10.1145/3308558.3313684

[B111] Wu W. Protopapas P. Yang Z. Michalatos P. (2020). “Gender classification and bias mitigation in facial images,” in 12th ACM Conference on Web Science, 106–114. 10.1145/3394231.3397900

[B112] Yang Z. Feng J. (2020). “A causal inference method for reducing gender bias in word embedding relations,” in Proceedings of the AAAI Conference on Artificial Intelligence (New York, NY), 9434–9441. 10.1609/aaai.v34i05.6486

[B113] Zhao J. Wang T. Yatskar M. Ordonez V. Chang K.-W. (2017). Men also like shopping: Reducing gender bias amplification using corpus-level constraints. arXiv preprint arXiv:1707.09457. 10.18653/v1/D17-1323

[B114] Zhao J. Zhou Y. Li Z. Wang W. Chang K.-W. (2018). Learning gender-neutral word embeddings. arXiv preprint arXiv:1809.01496. 10.18653/v1/D18-1521

